# Surgical resection of a giant cardiac angiosarcoma and reconstruction of involved right heart structures: A case report

**DOI:** 10.3389/fcvm.2023.1115962

**Published:** 2023-03-01

**Authors:** Andreea Blindaru, Alexandru Vasilescu, Andrei Danet, Oana Zimnicaru, Maximilian Cristu, Stefan Tudorica, Tudor Borjog, Oana Patrascu, Catalin Constantin Badiu

**Affiliations:** ^1^Department of Cardiovascular Surgery, University Emergency Hospital Bucharest, Bucharest, Romania; ^2^Department of Anesthesiology, University Emergency Hospital Bucharest, Bucharest, Romania; ^3^Department of Anatomopathology, University Emergency Hospital Bucharest, Bucharest, Romania

**Keywords:** cardiac angiosarcoma, surgery for cardiac angiosarcoma, primary cardiac tumor, surgical resection of giant cardiac angiosarcoma, angiosarcoma of the heart, case report

## Abstract

We present the case of a young woman without a medical history who presented with a giant right atrial, transtricuspid, and right ventricular mass and in a severe clinical state. Multimodal imaging raised the suspicion of primary cardiac angiosarcoma. Due to rapid hemodynamic and respiratory deterioration, we were forced to perform surgical removal of the mass with a concomitant reconstruction of the involved right heart structures, only 48 h after presentation. The postoperative course was uneventful, and the patient was discharged from the intensive care unit 2 days later. Radical surgical resection with reconstruction of the resected heart structures was the only possible salvage option for giant angiosarcoma, which led to hemodynamic instability. Followed by chemotherapy, this radical approach may prolong survival.

## 1. Introduction

Malignant cardiac tumors are scarce clinical entities with poor prognosis and a median survival time ranging from 6 months to a few years (1). Their occurrence in autopsy series varies between 0.001 and 0.28%, with angiosarcomas being the most common form (2). Aggressive local growth and metastasis are common features of angiosarcoma, and the primary site for metastases is represented by the lungs (3). Over the last decades, different treatment options have been proposed for dealing with angiosarcoma, unfortunately with poor results. This is likely related, on the one hand, to the complexity of the complete surgical resection of the tumor and the involved vital structures and, on the other hand, to the relative non-responsiveness of angiosarcomas to adjuvant therapies (1).

## 2. Case report

We report the case of an 18 years-old woman without a medical history who was admitted with symptoms of fatigue, dyspnea, and leg edema, which had started and worsened over the course of 3 weeks. The patient had no previous symptoms and no family history of malignancies. Upon admission, various diagnostic tests were conducted, including an electrocardiogram, echocardiography, computed tomography (CT), and magnetic resonance imaging (MRI). On admission, the patient’s vital signs were normal (heart rate 95 bpm, blood pressure 110/60 mmHg, and oxygen saturation 98–99%). Laboratory results showed normochromic normocytic anemia (Hgb 11,7) and thrombocytopenia (PLT = 75.000 μL). The electrocardiogram revealed sinus tachycardia and a minor right bundle branch block. Physical examination showed dyspnea and leg edema, but no heart murmur and no jugular venous distension. Echocardiography revealed moderately impaired RV function, almost normal LV function, and a large mass occupying the right atrium and extending partially into both caval veins (Supplementary Figure 1). The mass was also expanding through the tricuspid valve into the RV, obstructing the RV inflow and outflow tract. The anterior and posterior tricuspid leaflets were engulfed in the tumoral mass (Supplementary Video 1). MRI confirmed the echocardiographic findings and further described the mass enclosing a part of the right coronary artery. The MRI appearance of the mass was highly suggestive of angiosarcoma (small intralesional foci with hyperintense T1 signal and hyperintense T2 signal compared to the myocardium, postcontrast heterogenous enhancement with gadolinophilic tissue areas, and intratumoral necrotic inclusions) (Supplementary Figure 2 and Supplementary Video 1). A full body CT scan revealed multiple small pulmonary nodules, but no other secondary determinations.

Differential diagnosis of cardiac angiosarcoma must include hemangioendothelioma, intracardiac metastases (including metastatic angiosarcoma) as well as leiomyosarcoma, fibrosarcoma, or malignant fibrous histiocytoma.

To determine the therapeutic strategy, the patient was evaluated by a multidisciplinary team that included a cardiologist, oncologist, anesthesiologist, and thoracic and cardiovascular surgeon.

The patient was provided with psychological support to help her understand the severity of her diagnosis and the necessity of continuous treatment. It was our patient’s decision to continue with the treatment.

Due to severe obstruction of the tricuspid valve, the patient developed a low cardiac output state, leading to severe hemodynamic instability, and therefore, we were forced to perform urgent surgical resection of the tumor. After sternotomy and adhesiolysis, a right femoro-femoral and concomitant distal cannulation of the superior vena cava was performed. Antegrade cardioplegia was administrated, and the right atrium was opened by resecting the entire free wall. Intraoperatively, we found a giant mass (11/10 cm) invading the entire right atrium, including the free wall, one-third of the right coronary artery, the anterior and posterior tricuspid valve leaflets, the basal part of the RV free wall, and a significant part of the RV cavity (Supplementary Video 1). The mass extended also to the proximal parts of both caval veins. Extensive tumoral resection was performed. Approximately 7 cm of the right coronary artery had also to be excised, and accordingly, a right coronary bypass graft was necessary. After complete macroscopic resection of the tumor (Supplementary Video 1), the reconstruction began with the implantation of a valvular prosthesis. As two-thirds of the annular circumference was resected, the bioprosthesis had to be fixed by pledged sutures to the endocardium of the RV free wall and to the septal leaflet (Supplementary Video 1). The right atrial wall was reconstructed using a generous bovine pericardial patch, which was directly sewn to the prosthetic valve ring, the interatrial septum, and the caval veins (Supplementary Video 1). Weaning from CPB was uneventful, and the aortic-cross-clamp time and CPB time were 115 and 239 min, respectively. The early postoperative course was uncomplicated, and our patient was discharged from the ICU 2 days later. The postoperative TEE showed a normofunctional bioprosthesis, with a mean diastolic gradient of 1.3 mmHg, moderate RV dysfunction (FAC 24%), and mild LV dysfunction (Supplementary Video 1).

The diagnosis of high-grade angiosarcoma was confirmed by the histopathologic and immunohistochemistry examinations, which were positive for CD31, CD34, ERG (erythroblast transformation-specific-related gene), and Ki67 (Supplementary Figure 3).

Due to the presence of lung metastases and the R1 resection, following surgery, the patient underwent chemotherapy with ifosfamide (3,000 mg) and mesna (2,800 mg). Starting from the second cure, the chemotherapy was extended with doxorubicin (40 mg). At 6 months of follow-up and after completing six sessions of chemotherapy, the patient presented in a stable clinical state NYHA I, with normal LV function and mild RV dysfunction. Furthermore, there was no evidence of tumor recurrence, and the small pulmonary nodules could not be identified anymore in the follow-up CT performed at 6 months. Unfortunately, 2 months later, a 20/19 mm intracranial metastasis was identified on the brain CT scan, and after 1 month, the patient underwent surgical resection and started radiotherapy. The chronological order of clinical events is shown in Supplementary Table 1.

## 3. Discussions

Cardiac angiosarcoma is a rare entity in clinical practice, and thus, there is a lack of consensus guidelines on the management of this pathology. Cardiac angiosarcoma is often found in the right atrium, but it can also invade the right ventricle, the tricuspid valve, and the right coronary artery.

Patients are asymptomatic only if the tumor is discovered in a very early stage. As the tumor mass grows, it may exhibit not only varied symptoms such as dyspnea or chest pain but also hemoptysis and embolic events (4). Early-stage diagnosis should be managed by immediate surgical therapy, as several studies have demonstrated the best long-term survival after complete resection in small angiosarcoma (1, 4, 5). Furthermore, as reported by different groups, a preoperative endomyocardial biopsy may lead to complications without being a very sensitive method for angiosarcoma diagnosis. Accordingly, this procedure should not be mandatory (6). Nevertheless, early-stage cardiac tumors are diagnosed incidentally or, unfortunately, often overlooked.

Due to rapid infiltrating growth, in patients with advanced right-sided angiosarcoma, the clinical presentation may be dominated by complications deriving from the massive tumor (7).

Right-sided tumors are bulky, infiltrative, and tend to metastasize early, but they do not usually present congestive heart failure until late in the disease (8, 9). To accurately characterize the tumor location and size, as well as its possible distant metastatic lesions, non-invasive imaging methods such as echocardiography, CT, and MRI contribute essentially. Thus, even if surgery may be often challenging, good imaging may improve preoperative planning and reduce the risk of operative mortality (10). Furthermore, imaging can even postpone surgery in some patients and prefer preoperative chemotherapy as a possibility of shrinking the tumor mass (11). This approach was used by Blackmon and Reardon who report their experience with 15 operated angiosarcomas in patients with no heart failure or imminence of heart failure. They started with preoperative chemotherapy to shrink the tumor, followed by surgical resection. In their case, median survival was 27 months, with the longest-surviving patient alive at 9.5 years. Our patient developed heart failure due to obstruction of the tricuspid valve, and such an approach was not possible at the time of presentation (5).

As described by Yadav and Mangla (8) in their study, surgical resection with an R0 margin is associated with the best outcomes. In situations where R0 resection is not achieved, chemotherapy and radiation therapy after surgical resection may improve results.

Nevertheless, technically feasible, surgical resection still represents the gold standard and an important prognostic factor for angiosarcoma treatment. In our case, surgery was necessary to achieve hemodynamic stability and relieve the life-threatening obstruction. Similar to other reports where R0 resection was achieved only in approximately 25% of cases, in our patient, an R0 resection has not been demonstrated by the histopathologic examination (10). Nevertheless, even in cases where R0 resection is possible, there still remains a high risk for local recurrence or even the appearance of postoperative metastatic lesions (12). As recommended by other authors, adjuvant chemotherapy should be considered in all patients (10). Even if there are no clear recommendations or clearly designed protocols for radiotherapy or targeted therapy as adjuvants to surgery, it has been reported that they may improve survival, especially in patients with R1 resection (10, 13).

In our case, the decision to offer immediate surgery and postpone chemotherapy was driven by the hemodynamic instability, even if several vital structures were infiltrated. The surgical judgment was based on the fact that complete resection of these structures may not severely influence the right heart function, especially as they were already affected by the tumor infiltration. Hence, right ventricular contractility was not severely affected by the ventricular wall resection as the excised area was already akinetic before surgery. Furthermore, the resected coronary artery could be replaced by a venous graft, as already described in the literature, without influencing the contractility of the remained RV (14). The replacement of the resected tricuspid valve offered a stable structure to rebuild the right atrioventricular groove and the right atrial wall using a heterologous pericardium. Hence, we advocate for complete surgical resection of the infiltrated right heart structure s in this malignant pathology whenever a sufficient part of the right ventricle remains functional.

Recently, a combination of proton beam therapy and chemotherapy followed by adjuvant chemotherapy has emerged as a promising approach for patients with R1 resection of right-sided angiosarcoma. Mangla et al. (15) reported a survival time of 18 months after treatment completion in a young man. Taxanes have been found to have similar efficacy compared to anthracyclines, however, without the cardiotoxicity associated with anthracyclines. Although survival after proton beam therapy is encouraging, it is not currently available at our institution (15).

## 4. Conclusion

Taking into account that cardiac angiosarcoma is associated with a very poor prognosis, an early-stage diagnosis should be immediately treated with surgical resection and a meticulous postoperative follow-up. In advanced cases, preoperative imaging should help plan aggressive resection and reconstruction of the involved structures. Multidisciplinary teams must establish the applications and benefits of postoperative adjuvant therapy, which may improve long-term survival.

## Data availability statement

The original contributions presented in this study are included in this article/Supplementary material, further inquiries can be directed to the corresponding author.

## Ethics statement

Written informed consent was obtained from the participant for the publication of this case report. Written informed consent was obtained from the individual(s) for the publication of any potentially identifiable images or data included in this article.

## Author contributions

CB was part of the surgical team, reviewed and revised the manuscript critically for important intellectual content, contributed to the conception, and design and approved the final manuscript as submitted. AB was part of the surgical team, drafted the initial manuscript, contributed to the conception and design, and approved the final manuscript as submitted. AV was part of the medical team, reviewed and revised the manuscript, and approved the final manuscript as submitted. AD was part of the surgical team and contributed to data analysis and interpretation. OZ was the echocardiographist of the team and approved the final manuscript as submitted. MC was the video editor, reviewed and revised the manuscript, and approved the final manuscript as submitted. ST was part of the anesthesiological team and contributed to data analysis and interpretation. TB was part of the anesthesiological team and contributed to data analysis and interpretation. OP was the anatomopathologist and contributed to data analysis and interpretation. All authors contributed to the article and approved the submitted version.
