# Supplementary material for: Surgical resection of a giant cardiac angiosarcoma and reconstruction of involved right heart structures: A case report
Source: Front Cardiovasc Med. 2023 Mar 1;10:1115962. doi: 10.3389/fcvm.2023.1115962 (PMC10014811; doi:10.3389/fcvm.2023.1115962)
Supplement: Supplementary file 2 [file Data_Sheet_1.docx]

Supplementary Material

Surgical resection of a giant cardiac angiosarcoma and reconstruction of involved right heart structures: a case report.

Andreea Blindaru MDa†, Alexandru Vasilescu MDa†, Andrei Danet MDa, Oana Zimnicaru MDa, Maximilian Cristu MDa, Stefan Tudorica MDb Borjog Tudor MDb, Oana Patrascuc, Catalin Constantin Badiu MD, PhDa.

**Address for correspondence:**

Name: Andreea Blindaru

Full Postal Mailing Address: Department of Cardiovascular Surgery, University Emergency Hospital Bucharest, Splaiul Independentei 169, Bucharest 050098, Romania.

Telephone:+40741077337

E-mail: blindaruandreea@gmail.com

1. Supplementary figures and tables

1.1 Supplementary figures


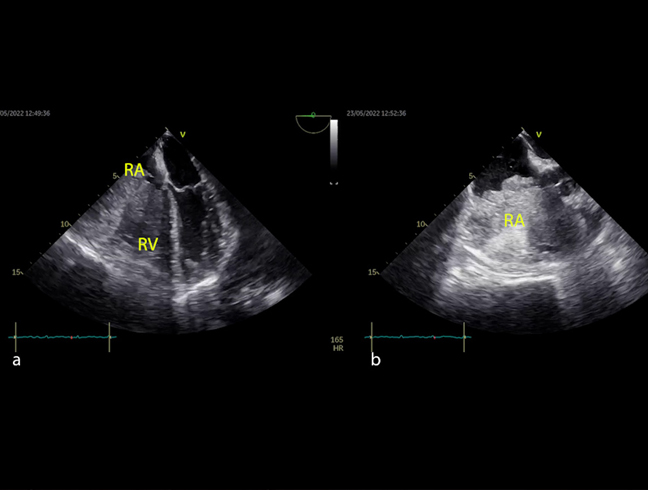


Figure 1


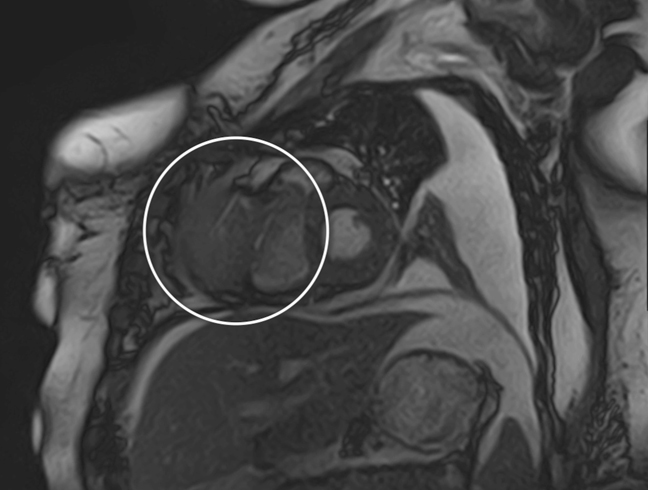


Figure 2


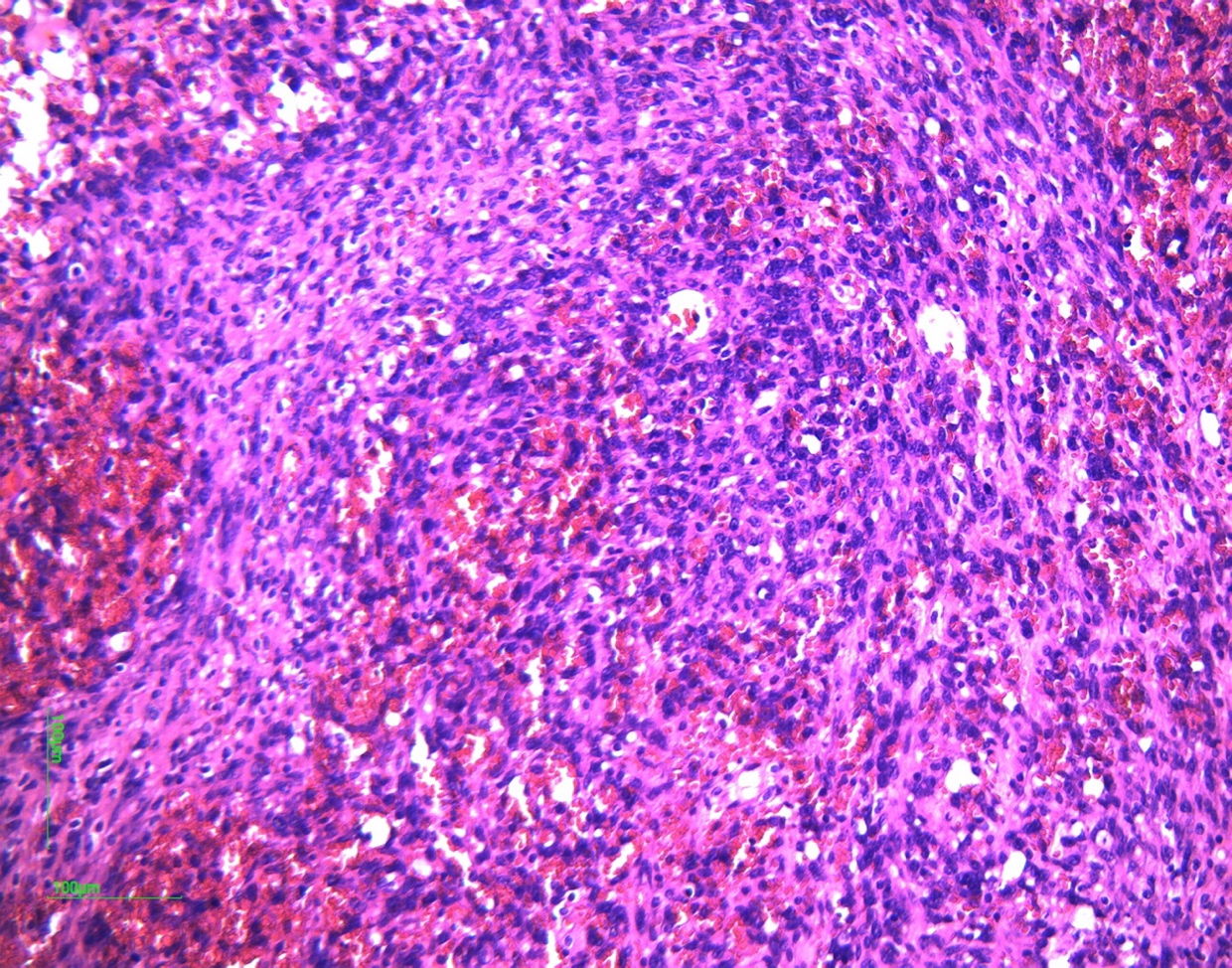


Figure 3

1.2 Supplementary tables

Table 1: Table Timeline

| Day 1 | An 18-year-old woman was admitted to the department of cardiovascular surgery for fatigue, dyspnea and leg oedemas.  Echocardiography revealed a large mass occupying the right atrium, extending into both caval veins and also expanding transtricuspidian, into the RV. |
| --- | --- |
| Day 2 | CT confirmed the echocardiographic findings. |
| Day 3 | MRI confirmed the echocardiographic findings and further described the mass enclosing a part of the right coronary artery.  MRI appearance of the mass was highly susceptible for angiosarcoma.  Heart Team evaluation. |
| Day 4 | Hemodynamic deterioration and surgical resection of the giant atrial mass. |
| Day 6 | Discharge from the ICU. |
| Day 12 | The diagnosis of high grade angiosarcoma was confirmed by the histopathologic and immunohistochemistry examinations. |
| 1 month after surgery | Initiation of chemotherapy. |
| 6 months after surgery | Follow-up CT scan without tumor recurrence |
| 8 months after surgery | Follow-up brain CT with intracranial metastasis. |
| 9 months after surgery | Resection of the cranial tumor and radiation therapy. |

Figure legends:

Figure 1: Preoperative transesophageal echocardiography

TEE depicting large isoechoic mass originating in the right atrial wall, with a broad base, that encapsulates anterior and posterior tricuspid leaflets, and protrudes into the RV.

a) Four chamber mid-esophageal

b) RV outflow-inflow

Figure 2: Preoperative cardiac magnetic resonance

CMR showing a large atrial mass, with transtricuspidian extension in the right ventricle, the right ventricular outflow tract, and also enclosing a part of the right coronary artery.

Figure 3: Histological image of the tumor

Proliferating spindle cells with cytological atypia forming immature vascular channel, CD34 positive (specific marker for endothelial cells) col HE 20x.

Abbreviations

RA - right atrium

RV - right ventricle

Video 1: Imagistic findings and intraoperative key moments.

The first screen shows preoperative transesophageal echocardiography imaging from four chamber midesophageal and RV outflow-inflow views, showing the tumor originating in the right atrial wall, with a broad base, that encapsulates anterior and posterior tricuspid leaflets, and protrudes into the RV.

The second screen shows cardiac magnetic resonance, showing a large atrial mass, with transtricuspidian extension in the right ventricle, the right ventricular outflow tract and also enclosing a part of the right coronary artery.

The first intraoperative imaging shows the giant tumoral mass. A thorough dissection is performed to divide the tumoral mass from the healthy tissue and to achieve a complete macroscopic resection. About seven cm of the right coronary artery had to be excised and accordingly a right coronary bypass graft was necessary. After complete macroscopic resection of the tumor, the reconstruction began by implantating a valvular prosthesis. As two third of the annular circumference was resected the bioprosthesis had to be fixed by pledged sutures to the endocardium of the RV free wall and to the septal leaflet. The right atrial wall was reconstructed using a generous bovine pericardial patch, which was directly sewed to the prosthetic valve ring, the interatrial septum, and the vena cava.

The last screen of the video shows postoperative transesophageal echocardiography depicting the resection of the tumoral mass, the reconstructed structures and the normofunctional bioprosthesis in four chamber midesophageal and RV outflow-inflow views.
